# Southern Dental Association—Fourth Annual Session

**Published:** 1872-09

**Authors:** 


AMERICAN JOURNAL

OF

DENTAL SCIENCE.

Vol. VI. THIRD SERIES—SEPTEMBER, 1872. No. 5.

### ARTICLE I.

### *Southern Dental Association—Fourth Annual Session.*

FIRST DAY—MORNING SESSION.

The fourth annual meeting of the Southern Dental Association was held in the large hall of the Exchange Hotel, Richmond, Va., July 30th, 1872. The meeting was called to order by President F. Y. Clark, of Savannah, Georgia. Prayer by Rev. C. C. Bitting, of the Second Baptist Church. Mayor Kelly was next introduced by the President, who, in behalf of the city of Richmond, extended to each member of the Southern Dental Association a most cordial greeting.

He was particularly gratified that Richmond had been selected as the place of assemblage for so honorable a body, and it was especially a pleasure to him to look around and greet the representatives of so many Southern States. He closed his address in his peculiar and happy style, and was warmly applauded by the members present.

The address of welcome was next delivered by Dr. Wm. W. H. Thackston, of Farmville, Va., who entertained the delegates and others present pleasantly for half an hour, extending a cordial welcome of Virginia to each and every member present.

Dr. F. Y. Clark, President, next read his annual address to the Association. He declared that an old Virginia welcome had just been received from the Chief Executive of the city—a pioneer of the profession—a man who about twenty-three or four years ago received from the first and then only dental college in the world the third diploma issued, making him now one of the oldest dental graduates living; and were it not for the duties resting upon the President, and the business matters connected with what he had to say he would not add one word to the able and eloquent remarks to which the Association had just listened. He felt proud for many reasons that the meeting over which he presided was held within the proud old historic city of Richmond. With many others he had long wished to meet the profession of Virginia, and last year, at the Charleston meeting, it was decided, "Mahomet like, as the mountain would not come to us, to go to the mountain," knowing that we would receive the world-wide old Virginia welcome, known nowhere so well as in Richmond, the cradle of its birth.

The address next alluded to the name of the Association and the fear on the part of the speaker that some might think there was antagonism between this and the American Association. But there was no such thing. The name might sound a little sectional, and no doubt some such word as national would become the Association better; but the great object was to secure an association that would hold its meetings in our midst, so that the members of the profession in each of the respective States could accordingly assemble together and have the advantage of an associated body. The American Association did not give the Southern Association this advantage, its meetings being, as a general thing, held in the North previous to the organization of the Southern Association, thus obliging southern representatives to make long and expensive trips, which many, after the war, were unable to make.

The address also urged the necessity of a correct report of the proceedings of the Association, and touched upon the feasibility of publishing a dental journal.

The transactions of the Southern Dental Association and discussions at its third annual meeting, held at Charleston on the 12th, 13th, 14th and 15th of April, 1871, were then read by the Secretary. This engaged the attention of the meeting for an hour.

It being announced that Dr. Robert Arthur, of Baltimore, one of the oldest and most well-known members of the profession, was in the hotel, a committee consisting of Drs. Thackston, Thompson and Grant, was appointed to wait upon and invite him to a seat in the hall during the deliberations of the body.

On motion of Dr. Thompson, of Fredericksburg, a recess of ten minutes was taken in order to receive applications for membership.

After recess, Dr. Thompson, from the Committee on Membership, reported the following applicants, who were duly elected: Drs. A. G. Bouten, Baltimore; James Johnston, Staunton, Va.; Jud. B. Wood, Richmond; William A. Mills, Norfolk; J. Hall Moore, Richmond; G. G. Wayt, Richmond; John Mahoney, Richmond; T. B. Legare, Orangeburg, S. C.; John W. Scribner, Charlottesville, Va.; George F. Keesee, Richmond; R. W. Hudson, Richmond; William B. Pleasants, Richmond; F. A. Jeter, Richmond; and G. G. Davidson, Lexington, Va.

On motion of Dr. W. W. H. Thackston, Dr. Robert Early, of Lynchburg, was elected an honorary member of the Association.

Dr. Grant from the Executive Committee, submitted a report declaring that the business assigned to that Committee had been discharged, that the committee had failed to employ a stenographic reporter, and that all railroads would return delegates to the Convention free of charge. Adopted.

On motion of Dr. Thompson from 8 to 10 A. M. daily was set apart for clinical operations; and for business from 10 to 2 P. M. and from 3½ to 6 P. M.

Adjourned until 3½ P. M.

AFTERNOON SESSION.

The Association reassembled at half-past 3 o'clock—President Clark in the chair.

On motion of Dr. Grant, a committee was appointed to examine and report upon new appliances and inventions that may be prepared for the notice of the profession.

The Chair named the following gentlemen as the committee: Drs. James Johnston, of Staunton, Va.; Dr. E. Floyd, of Fayetteville, N. C.; and F. A. Jeter, of Richmond.

Dr. Samuel A. White, from the Committee on Publication, submitted a report on publication, accompanying which was a note from the chairman regretting that he was unable to be in attendance upon the sessions of the body.

Dr. John G. Angell, from the Committee on the Journal, reported favoring the publication of a monthly journal, but stated also that the committee were moving cautiously with the work. He is detained from the Association by indisposition. Adopted.

There was no formal report from the Committee on Dental Education, of which Dr. J. P. H. Brown was chairman, he being prevented from attending the sessions of the body. A communication from that gentleman, however, giving his views as to dental education and that which should be required of a dental student, was read and received.

Dr. F. J. S. Gorgas regarded this as a most important subject. He thought that the profession of teaching was rather one of love than remuneration. He gave an interesting account of the operations of the college in which he is a professor, (Baltimore Dental College,) and declared that he could point with pride to the most eminent in the profession who are graduates of this college. He hoped that the standard would be raised, and to the whole profession of the South he suggested the endowment of a dental college which would elevate the standard in that land; and but little money would be required to place one of the two institutions of this kind, already in the South, where they should properly stand. The Baltimore College had prior to the war the greatest number of students. The Northern students do not count more than three or four, the large majority being from the South, save two or three from Europe.

Dr. Clark said that he rose to speak upon a subject which he regarded as one of the most important that could come to the attention of the Association. He thought that all of our colleges ought to be turned into one or two schools. He spoke of the trouble of issuing honorary diplomas, and how the difficulty was sought to be remedied in Georgia by the adoption of a resolution throwing the whole of the responsibility on the State societies. He thought that this would be the means of avoiding future trouble. Many students had received diplomas who were not entitled to them. He also spoke earnestly of the importance of centering all efforts on one or two schools—thus recognizing some one particular school.

Dr. Gorgas said that only one honorary diploma had been issued from his college since 1860; that his college had made it a rule not to issue these diplomas unless in cases of special merit.

[At this point the report of the Executive Committee was discussed and the thanks of the Association returned to the committee for the faithful performance of their work.]

Dr. Arthur, of Baltimore, appearing upon the floor, was introduced to the Association by the President, Dr. Clark. Dr. A. returned his thanks for the honor paid him, and took his seat with the members.

The report of the Committee on Histology and Microscopy, prepared by Dr. S. P. Cutler, chairman, of New Orleans, was read, as well as a communication from Dr. E. A. Hermon, chairman of the Committee on Operative Dentistry, on that subject.

Dr. Thompson moved that in lieu of this last report, the work of Dr. Arthur, of Baltimore, on operative dentistry be taken up, and that he be requested to act as chairman selecting such portions for discussion as he might think best

The work was then made the special order for Wednesday morning at 10 o'clock.

On motion of Dr. Grant the regular business was suspended, and the Committee on Membership presented the name of Dr. W. Leigh Burton, of Richmond, who was duly elected.

On motion of Dr. Thompson the regular physicians of the city were invited to seats upon the floor.

On motion of Dr. Grant, Dr. W. Leigh Burton read a paper which he had prepared on the subject of "fracture and subsequent reunion of a superior left central incisor or deciduous tooth."

This subject was then discussed by Dr. Clark and others.

The Chair appointed the following committee on clinical operations for Wednesday morning from 8 to 10 A. M.: Drs. Johnston of Staunton, Gorgas of Maryland, and Pleasants of Richmond.

Adjourned.

SECOND DAY—MORNING SESSION.

The Southern Dental Association reassembled at 10 o'clock Wednesday morning. Prayer by Rev. Moses D. Hoge, of the Second Presbyterian Church.

The Chair announced that in accordance with the resolution passed yesterday the Association was ready to hear from Dr. Arthur, chairman of the Committee on Operative Dentistry.

Dr. Arthur said that he was at any time ready to meet and discuss with any person his theory and to sustain his views. If he could be shown that he is in error he would be willing and ready to acknowledge it. His theory, he acknowledged, is a fundamental change of practice. He had taken great pains to explain this method in Baltimore in the most elaborate manner, and yet he was not aware of one single person in that city practising it, though no one seemed to object. He had looked for opposition, and did not hope that his views would be immediately adopted. It has been clearly stated that it is a preventive system. He had come to these conclusions after great deliberation, for it seemed to be a great piece of presumption for one man to put his views in opposition to all others. In regard to the separation of sound teeth, he had to say that the system should be of the most thorough kind, as well as in the practice of dentists generally. One fact he desired to impress upon each member present, which was: that, even considering the advance in this art, the practice of dentistry, even in this enlightened age, is miserably defective; and there are many in the profession who are decidedly an injury to it. Sometime since a lady of seventeen years placed herself in his care. Her teeth had been very recently attended to by a dentist who was a good operator, and who had declared that her teeth had received all the requisite attention. He had made twenty-five fillings, besides removing seventeen decays. In less than one year the nerves of many of her teeth would have been entirely exposed. He thought it the deep obligation of the profession to be most careful in their operations and practice. He thought the most remunerative practice was to destroy the teeth in place of saving them. The time has come when it is the duty of the profession to correct these errors; and when an individual goes to a dentist and places himself under his care it is his bounden duty to examine carefully every one of his teeth, and satisfy himself as to their condition, and to be held responsible for the result.

Dr. Arthur, continuing his remarks, spoke of the manner of separating the teeth, and alluded to the use of the file as being a most objectionable instrument. The mode adopted by him in separating the teeth was the use of the chisel. By this means the file was dispensed with. It was a very laborious operation, but rather a relief to the patient. He had stated at that time that advancement would be made, and he was gratified to find it. He alluded to a new drill, and declared that it was indispensable to him. He had devised a number of wheels, which were adapted by him to the engine used. The greatest difficulty in using these instruments, or drills, is experienced by the rapid speed at which the machine moves, the utility of the instrument in a measure being destroyed. In order to use the wheels when they are large in diameter great care must be taken, as this high rate of speed will render accidents liable to occur. The speaker next alluded to the advantage of using the small chisel, and cutting from the gum downward when the other instruments had been applied. An objection to this method had been urged by some that the operation between the teeth is a painful operation to the patient. This he denied, and said in this connection that this method should be used in the earliest stage of decay. A dentist ought to be able at a glance at the teeth of a child of twelve years to tell the extent of decay. In cases in which caries attack the proximate surface of the incisor teeth before the twelfth year, not five per cent. of the individuals suffering the attack will escape caries of all the proximate surfaces of the teeth except the lower incisors.

Dr. Moore, of South Carolina, returned his thanks to Dr. Arthur for his very valuable work and his remarks, and desired to ask him how the process of hardening is accomplished.

Dr. Arthur stated that this subject was fully explained in his work.

Dr. Thackston had read with inexpressible satisfaction the valuable work of the gentleman who had just taken his seat, and he was prepared to say that the system of treatment advised by him was the most valuable and the best that could be devised for society. He had seen nothing in all he had read in criticism upon the Doctor's work that invalidated the force of his arguments, and it was an infinite pleasure to him to welcome it to the aid of the profession. He said that the operation of filing is liable to accident. At one period of his life he endeavored to protect the enamel, but it had resulted in failure in many instances. He took occasion also to say that the failures in dentistry were subjects worthy to be discussed by the Association.

He closed by saying that if Dr. Arthur's book was carefully examined the objections found in the journal criticisms generally would be untenable. He hardly knew what to say in the presence of Dr. Arthur, but were he away, certainly his heart would be let out and his praises sounded for such a deserving volume.

Dr. Bond: Will Dr. Arthur's method preserve all classes of teeth?

Dr. Arthur: I know of no exceptions.

Dr. Wright of South Corolina, said he had never had a conversation with Dr. Arthur, yet he felt as if he had known him all his life. He would like to ask him many questions — one, Whether or not during pregnancy with the female patients pain would be intense by this treatment.

Dr. Arthur, replying, said that he had met with no difficulty on that score, for the system is not at all painful, and for that reason it possessed another advantage.

Dr. Moore asked whether or not Dr. Arthur had universal success in treating by his method that class of teeth known as consumptive teeth, or, in other words, the long, parallel teeth?

Dr. Arthur said that he never felt certain until he had made an examination of the teeth after the operation had been performed. While no unfavorable result had appeared in cases of this kind treated by him, yet he fully appreciated the difficulties encountered and intimated by the question.

Dr. Clarke added his testimony to that of the other dentists. He had read nearly every work on dentistry, but in this work of Dr. Arthur's the subject was so well treated, and the "bull taken by the horns," if he might use such an expression, that his heart was rejoiced, and the profession had been placed under lasting obligations for the method therein suggested. He then recited an instance of a lady who had been much pleased with the method contained in a work which he had given her to read. She declared that she had faithfully read it, and would hold him responsible for his treatment of the teeth of her children; that as to the cost of the requisite attention she did not care, the one object of saving her children's teeth was all that she desired. In regard to "consumptive teeth," he believed them to be the easiest class to be dealt with. He wanted to avoid any cavity where food could be deposited. The egg-shaped burr, he said, would cut away the decay above, and when that is out there is no shoulder. The action of the tongue will serve to keep the teeth cleansed. In filing, unless they could get the deposit away from the teeth, decay would go on. He thought he could not do any member of the profession a greater service than to recommend to each to get the work and place it in the patient's hands.

Dr. Bond: Are we justified in filing the teeth to a shape like saw-teeth in order to prove that exposed dentine will not decay?

Dr. Clark replied that he thought that the day of filling had passed. He opposed it in every way, and believed Dr. Arthur's chisels were better, and his late method the best. He could not see the necessity of using a file in that method.

Dr. Bond had seen teeth filed in the saw-shape by men in his State who called themselves first-class operators, and he thought it outrageous.

Dr. Ford said that he thought Dr. Bond was a little skeptical; only, however, on account of the different patients he had treated. This skepticism would be removed when the teeth were examined after operation. He thought it was applicable in all cases.

Dr. Moore said he was not altogether satisfied about his "consumptive teeth"; that it was wrong to separate such teeth. He believed the teeth would certainly move afterwards, and therefore the rule would not apply in these cases. If teeth are of such a nature that tape charged with any material for cleansing them can be used, it is well, but very difficult to get the patient to use this cleansing method. He had never attempted to separate the teeth of children—that is, by filing. While the root is in process of making the pulp has as much as it can do to fill out the tooth; and he thought the calcification was carried on from some other part of the body.

Dr. Clark remarked that the last speaker was laboring under a mistake. He must be assured that decay is going on before the separation is attempted; for if not, it would be like given medicine to a well man. The object, he contended, was not to remove the sound substance unless there was decay.

Dr. Thackston referred Dr. Moor to the fact that in childhood, in youth, and in advancing years, the strength of nature was greater than with adults, and that with a child's tooth there was less calcification to be accomplished, and the surface of a child's will repair far more readily than with the tooth of an adult. Even our muscles and our bones at that period are undergoing a change, and are much more easily reknit. And so it is with the teeth of a child.

Dr. Moore wanted facts on this theory. He wanted to know what was going on at the root while the surface was being calcified, and from what source the lime would be obtained to effect the calcification, it being required to complete the root?

In reply, Dr. Arthur said the supply of lime contained in the blood is always in excess, and when there is an increased demand for lime it is applied by nature to that point where it is most needed. He deemed it most advisable to make the separation at an early period in life.

Dr. Ford to Dr. Clark: What are the signs of decay?

Dr. Clark: Discoloration is not the only sign of decay. The blue tinge and the white tinge are also signs. The latter is, in my opinion, the worst.

Dr. Thackston: Were you to have a patient of lymphatic temperament, aged ten, eleven, or twelve years, with caries presenting on the proximate surface of the inferior incisors, would you not from that condition of these inferior incisors expect certainly to find decay upon the proximate surfaces of the molar and bicuspid teeth?

Answer—I certainly should.

Dr. Arthur said that in this system of practice, its highest features are to be applied, and if the decay touches the enamel and goes through, it is very difficult to arrest it, and you are frequently obliged to make the separation wider than you would under other circumstances. In some intances it is most difficult to detect the decay. The failures that have occurred in his hands have been when the caries had gone so far that it had to be removed after the separation had been made. If he sees a patient of a certain temperament whose teeth will decay at every point where they touch, why shall not the remedy be applied and the teeth separated? Hence the reason and the importance of a general view of this matter and application of the method at an early period in life.

Dr. Clark: If you find the decay in one member of the family, do you not look for the same in others?

Dr. Arthur: Not always. I had a case recently where a girl's teeth had every appearance of health; saw no appearance of decay, and hence I made no separation. The teeth of her brother, one year younger, were decayed at all the usual points of contact. I do not use the file except in urgent cases.

Dr. Thompson said he believed that the time could not be far distant when this plan of Dr. Arthur's will be adopted generally by the progressive men of the profession.

Dr. Clark: Did I understand you to say that dentine has more power to resist decay than enamel?

Dr. Thompson: Yes, sir.

Dr. Clark: Nature is the best criterion we have. What did nature place that enamel on the outside for, in place of the dentine? No, sir. Nothing can be better to preserve the teeth than the enamel.

Dr. Moore contended that that theory overthrew Dr. Arthur's views entirely.

"No, sir," replied Dr. Clark. "If Dr. Arthur tells me that dentine is superior to enamel, then I take issue with him and tell him he is wrong, for I take nature in preference to any such theory as that.

Dr. Thompson replied that a tooth that was isolated he never filed, but only in the case of abraded enamel.

Dr. Arthur said it was only in certain cases that his theory applied. In some instances the dentine has a greater power of resistibility than the enamel. He remarked that he had never made the sweeping assertion that dentine was more durable than enamel. The enamel, under the same circumstances, will not resist decay as well as the dentine, but after the enamel is attacked by decay it has no power of recuperation. The dentine has.

Here the debate closed.

At the conclusion of Dr. Arthur's remarks, Dr. Thompson, from the committee on membership, reported the names of Drs. J. R. Woodely, Joseph Woodward, and G. W. McElheny.

Dr. Grant, as the presiding officer of the Virginia Dental Association, extended an invitation to the Southern Dental Association, which he had received, for a trip up the canal and a collation at the Water Works. The invitation was accepted.

The President appointed the following committee to prepare suitable resolutions in respect to the memory of Drs. Reynolds and Roderigues—viz.: Drs. Moore, Lowrance, and Thackston.

The following resolution was also adopted :

"*Resolved*, That a committee of three be appointed by the Association to take into consideration, and recommend the adoption of some appropriate plan for the erection of a suitable monument sacred to the memory of Dr. Chapin A. Harris, of Baltimore."

Adjourned until 3½ P. M.

AFTERNOON SESSION.

The Association met again at 3½ o'clock—President Clark in the chair.

The Chair appointed the following committee to consider the subject of a monument to be erected to the memory of Dr. Harris, of Baltimore: Drs. H. M. Grant, of Virginia;

W. S. Brown, of South Carolina, and A. F. Bignon, of Georgia.

Dr. Gorgas, ot Baltimore, read the report of the Committee on Histology and Microscopy, which was received, and a vote of thanks extended to Dr. Cutler for its preparation, and to Dr. Gorgas for its reading.

The Association, on motion, next proceeded to the selection of a place for the next annual meeting.

Dr. Haip moved that Savannah be selected.

Dr. Moore suggested Raleigh.

Dr. Thompson thought that some point in Maryland should be selected, and moved that the Association hold its fifth annual meeting in the city of Baltimore, where the science is carried to such a degree of perfection.

Dr. Moore withdrew his nomination of Raleigh.

Dr. Wright nominated Cave City, Ky., but afterwards withdrew his motion.

Dr. Ford opposed Baltimore, and suggested Memphis or Louisville, but finally nominated Nashville. So far as he was concerned he was five hundred miles from anywhere, his home being in Atlanta.

Dr. Clark favored Baltimore, but insisted, if the Association went to Georgia, the meeting must be in Savannah He was willing, however, to give up Savannah if it would be decided to go to Baltimore.

Dr. Grant said that if there was anything in his heart next to the sustenance of his wife and children it was his desire to elevate his profession, and he therefore thought that this end could be accomplished by going to Baltimore The following year it would be well to go to St. Louis.

Dr. Gorgas assured the Association that if Baltimore was selected he for one would do all he could to give the Association a cordial welcome.

Atlanta was withdrawn, and, on motion, the President was requested to cast the vote of the Association. He did so, selecting Baltimore as the next place of meeting. The time of meeting was then decided to be the last Tuesday in [illegible] 10 A. M.

Dr. Thompson, from the Committee on Membership, presented the name of Dr. A. F. Bignon, of Georgia, who was duly elected.

The report on mechanical dentistry was read by Dr. Ford, and received.

Dr. Mills, of Norfolk, gave his views upon aluminum work.

Dr. Sprinkle said he had been using the aluminum casting for about eighteen months, the process by hydrogen gas, and that he liked it very much. He had eight patients who were wearing these castings, and they seem to be well pleased with them.

Dr. Gorgas, from the Committee on Dental Literature, submitted a lengthy report from that committee, which was read and received.

It gives a brief summary and review of the dental periodical publications of the country and of works relating to dental science that have appeared since the last annual meeting. The report also suggests that the Association take the *American Journal of Dental Science* under its charge and patronage. The discussion on dental literature was postponed until to-morrow.

The chair appointed the following committee on clinics for Thursday, from 8 to 10 A. M.: Drs. Bouton, of Maryland; Ford, of Georgia, and Thompson, of Virginia.

Adjourned until 10 o'clock Thursday morning.

In the evening the members of the Southern Dental Association, by invitation, visited Riverside Park and enjoyed a ride up the canal. On their return they stopped at the water works, and were handsomely entertained by the Virginia State Dental Society. Speech-making, toasting, and having a good time generally engaged attention until a late hour, when they returned to the city charmed with their entertainment.

### THIRD DAY—MORNING SESSION.

Pursuant to adjournment the Association met at 10 o'clock, President Clark in the chair.

After the reading of the proceedings of Wednesday, in which Dr. Arthur was reported as saying that it was more profitable to destroy the teeth than to preserve them, President Clark called upon that gentleman to explain himself, if he had really made the remark attributed to him.

Dr. Arthur: I meant that the practice of dentistry is greatly misunderstood by the public. The thorough treatment of the teeth in the case of any individual demands many difficult as well as simple operations. If the merely simple operations, occupying conspicuous places in the mouth are performed, they can be done with great rapidity. It is more profitable to attend to twenty people in this way than to one thoroughly. But the result will be that the teeth of any one treated in this way will suffer, and many of them eventually be destroyed. But it is easy to satisfy such individuals that the result is unavoidable. Such kind of practice, unfortunately, is too common. It is time that the public should understand that the preservation of the natural teeth are within the resources of dental surgery properly applied, and in any instance where a child is entrusted without restrictions in the hands of a dentist, the loss of any of its teeth must be attributed either to incompetency or unfaithfulness on the part of the dentist.

The committee appointed yesterday for the purpose of devising or suggesting some means to this Association to raise a fund for the purpose of erecting a suitable monument to the memory of the late Professor Chapin A. Harris, do respectfully present the following suggestions, namely:

That for this purpose every member of this Association and the presiding officer of every State Dental Society be requested to act as a general committee to solicit subscriptions of one dollar each from every dental practitioner in their vicinity.

The committee also recommend the appointment by the Chair of a treasurer to take charge of the fund to be raised for this purpose. Adopted.

The committee appointed to examine new improvements and appliances presented a report, which was read and adopted.

The committee appointed to prepare suitable resolutions to the memory of Dr. William Reynolds, of Columbia, S. C., presented a report; which was read.

The same committee also presented appropriate resolutions to the memory of Dr. B. A. Rodrigues, of South Carolina, which were agreed to.

A resolution was presented by Dr. Thompson, expressing the sympathy of the meeting at the death of the consort of Dr. Clark, President of the Association, which was agreed to, and the same ordered to be spread upon the minutes of the Association.

A number of bills were presented by the Executive Committee, approved, and ordered to be paid.

A number of letters were also read from gentlemen who were unable to be in attendance upon the sessions of the Association.

Dr. Ford presented the following resolutions:

"WHEREAS it is the earnest desire of this Association to do everything in its power to elevate the standard of our profession throughout the world, and more especially within the borders of our own Southern States, that territory being our peculiar charge; and

"WHEREAS, we at present believe that the education of students in our dental colleges as at present pursued, according to the existing organization of these institutions, is deficient, principally on account of the paucity of pecuniary means; therefore be it

"*Resolved*, That this Association deems it advisable to endeavor to raise the requisite pecuniary means wherewith to endow one of our Southern Dental Colleges, whichever may in the judgment of this Association best subserve their interests.

"*Resolved*, That in furtherance of this object, the President be requested to appoint a committee, to consist of one from each of our Southern States, the President being *ex-officio* chairman, the duty of whom it shall be to frame some feasible plan to carry out the objects of these resolutions, and present the same for the action of this Association at its next annual meeting; and in the meanwhile each member of said committee shall bring the matter prominently before every dentist within the limits of his own State, and solicit subscriptions therefrom for the formation of an endowment fund; report the amounts to the chairman, who in turn shall report the total amount to the Association at its next annual meeting."

Dr. Clark objected to one clause in the above resolution. He wanted some guarantee as to the use of the money, and with that objection removed he would support the resolution and work with a vim to obtain the desired object. He thought that it behooved this Association to look into so important a matter and to take hold of one of the colleges in the South and let it be a credit and an honor to the profession.

Dr. Ford said the resolutions were crude, he knew. He didn't pretend to be *au fait* in such matters, but the chief object he had in presenting the resolutions to the Association was to bring it to the attention of the body.

Dr. Gorgas felt some hesitation in expressing an opinion, as he was connected with one of the colleges alluded to But he thought it would be well to have the committee appointed and let them decide upon some plan and make some definite proposition.

Dr. Moore, of South Carolina, thought the Association was able to support one college by sending all of their students to it.

Dr. Ford wanted this to be a certainty. He wanted an endowment for the college and so free from pecuniary embarrassment that when a student is graduated it should be upon his merits. He also desired poor boys who might wish to study dentistry to have the privilege of entering this college when they had acquired a sufficient academic education to do so.

Dr. Floyd moved as a substitute for the resolutions that a committee of five be appointed, one member from each State, to draft resolutions appropriate to this subject, and bring the same before the Association in the afternoon.

Dr. Thackston said he had been for a long time associated with dental colleges, and had a wide experience in the results of such colleges. He submitted as a general observation that the profession generally had made an error in the magnifying of dental colleges; that the profession is not able to sustain a college in every capital city in the Union. He thought the large number of dental colleges in the northern cities was over-doing the thing. He thought one well established college in the South could be sustained; that if the profession would concentrate upon one of the two colleges in the wide-spread limits of the country, it would be sustained. He didn't believe that the South could sustain two; that it was impracticable, in his judgment, for the establishment of a dental college in New Orleans when there was one in Baltimore. He thought it might be well to combine the two colleges, and then for the profession to unite upon and support it. He concluded by deprecating the establishment of another dental college while two already existed in the South.

Dr. Moore, of Richmond, suggested that Dr. Ford should withdraw that portion of the resolution in relation to raising the funds.

Dr. Ford said he wanted the resolutions amended if the Association thought it best, but he didn't propose to withdraw any portion.

Dr. Floyd's substitute was agreed to, and the Chair appointed the following committee: Drs. Floyd, Thackston, Jeter, Brown and Ford.

Dr. Moore, of Richmond, presented the following resolution, which was adopted:

"*Resolved*, That there be added to the standing committees of this Association one to be designated the Committee on Dental Improvements and Appliances, whose duty it shall be to carefully examine and practically test during the interval between the annual sessions of the Association, all new appliances which may be offered to the profession, and report fully upon their merits, recommending for adoption and use such as they may find really useful and valuable."

By Dr. Thompson: A resolution of thanks to Colonel Carrington for the use of the hall in which the sessions of the body were held, and to the press for the excellent reports of the proceedings. Agreed to.

By Dr. Clark: A resolution of thanks to the Virginia State Dental Association for their hospitality. Agreed to.

The Treasurer submitted his report, which was read and approved.

The Association next proceeded to the election of officers for the ensuing year, with the following result:

*President.*—Dr. H. M. Grant, of Abingdon, Va.

Dr. Grant said he scarcely knew how to thank the Association for so great a compliment, and he appreciated the honor all the more for the reason that the position was unsought by him. He declared whatever talent he might have he would devote to the interest of the Association and to the elevation of the profession; and in concluding his graceful address of thanks hoped that each member of the body would lend his aid not only to the advancement of the profession, but also to bear with his own many short-comings.

*First Vice-President.*—Dr. F. J. S. Gorgas, of Baltimore, Md.

*Second Vice-President.*—Dr. T. T. Moore of Columbia, South Carolina.

*Third Vice-President.*—Dr. A. C. Ford, of Atlanta, Ga.

*Corresponding Secretary.*—Dr. O. J. Bond, of Marion, South Carolina.

*Recording Secretary.*—Dr. J. F. Thompson, of Fredericksburg, Va.

*Treasurer.*—Dr. H. A. Lowrance, of Athens, Ga.

The Association then adjourned until half past 3 o'clock.

### AFTERNOON SESSION.

The Association was again called to order at 3½ P. M.—Dr. Clark (president) in the chair.

The chair announced the first business in order to be the installation of officers elect of the Association. In accordance therewith he appointed Drs. Thompson, Wayt, and Bignon a committee to wait upon Dr. Grant, president elect, and escort him to the hall. This duty was performed, and Dr. Grant escorted to the presidential chair. The other officers were also duly installed.

Dr. Grant, on taking the chair, announced the following *Standing Committees* for the ensuing year:

*Executive Committee.*—Dr. W. W. H. Thackston; John Mahony, Virginia; F. J. S. Gorgas, A. G. Bouton, Md.; A. C. Ford, Georgia.

*Membership.*—Drs. H. J. Royal, Georgia; O. J. Bond, South Carolina; James Johnston, Virginia.

*Publications.*—Drs. F. J. S. Gorgas, Maryland; James F. Thompson, Virginia; H. A. Lowrance, Georgia.

*Dental Education.*—Drs. E. Floyd, North Carolina; Judson B. Wood, Virginia; James Knapp, Louisiana.

*Physiology and Surgery.*—Drs. S. P. Cutler, Mississippi; J. C. McAuley, Alabama; S. A. White, Georgia.

*Histology and Microscopy.*—Drs. William H. Atkinson, New York; B. F. Arrington, North Carolina; E. M. Allen, Georgia.

*Dental Chemistry.*—Drs. George H. Winkler, South Carolina; John G. Angell, Louisiana; Samual Rambo, Alabama.

*Dental Therapeutics.*—Drs. G. T. Barker, Pa.; W. C. Wardlaw, Georgia; H. J. Royal Georgia.

*Operative Dentistry.*—Drs. F. Y. Clark, Georgia; S. J. Cobb, Tennessee; T. T. Moore, South Carolina.

*Mechanical Dentistry.*—Drs. S. G. Holland, Georgia; G. F. Keesee, Virginia; E. A. Herman, Tennessee.

*Dental Literature.*—Drs. G. F. S. Wright, South Carolina; T. B. Legare, South Carolina; J. B. Patrick, South Carolina.

*Voluntary Essays.*—Drs. W. H. Shadoan, Kentucky; W. H. Burr, Georgia; J. W. Scribner, Virginia.

*Dental Appliances and Improvements.*—Drs. F. A. Jeter, Virginia; A. F. Bignon, Georgia; W. B. Pleasants, Virginia; G. W. McIlhenny, Georgia.

Dr. Thompson presented the following resolution, which was agreed to:

"*Resolved*, That a vote of thanks be tendered to the retiring officers of this Association for the very able and efficient manner in which they have discharged their duties."

The chair appointed the following gentlemen as delegates from the Southern Dental Association to the national body, which meets on Tuesday next at Niagara.

Drs. T. T. Moore, South Carolina; G. G. Wayt, Virginia; Judson B. Wood, Virginia; Samuel Rambo, Alabama; O. J. Bond, South Carolina; W. H. Thackston, Va.; James F. Thompson, W. S. Brown, Georgia; G. F. S. Wright, South Carolina; James Johnston, Virginia; H. C. Jones, Virginia; E. Floyd, North Carolina; H. Lowrance, T. B. Lagare, South Carolina; G. W. McIlheny, Georgia; S. J. Cobb, Tennessee; John Mahoney, Virginia; F. J. S. Gorgas, A. G. Bouton, Maryland; A. C. Ford, Georgia; F. Y. Clark, Georgia; R. N. Hudson, F. A. Jeter, Virginia.

Dr. S. J. Cobb, of Tennessee, on motion of Prof. Gorgas, was invited to read an essay on teething; for which a vote of thanks was extended to Dr. Cobb.

Dr. Clark presented the following resolution, which lies over under the rules:

"*Resolved*, That the name of National Dental Association be substituted for Southern States Dental Association."

On motion of Dr. H. A. Lowrance, a vote of thanks was tendered Dr. M. Bissell, an old practitioner of South Carolina, for exhibiting a beautiful set of teeth carved by him out of ivory.

On motion of Dr. Clark, Dr. Thackston, of Farmville, Va., was elected an active member of the Association. Dr. Thackston has heretofore been an honorary member.

The committee appointed at the morning session of the Association on the subject of dental education, of which Dr. Thackston was chairman, presented the following as the result of their deliberations, which was read and adopted.

The president, Dr. Grant, will appoint the committee at his earliest convenience, and notify the secretary and chairman of each standing committee of such appointment:

"*Resolved*, That in furtherance of the object of this resolution the President of this Association be requested to appoint a committee of one from each of the Southern States, the President of this Association, *ex officio*, being chairman, whose duty it shall be to correspond with the faculties of the Baltimore Dental and New Orleans Colleges in relation thereto, and ascertain from them upon what terms either or each of them will consent to a reorganization of their faculties or an amalgamation of the two colleges, and in consideration of a compliance with either of these terms the college so reorganized will be adopted and endowed by this Association; that the said committee be instructed to bring this enterprise prominently before the members of the profession in their respective States, and at the same time request of their brethren what amount they will subscribe towards this endowment, and report to the Association the whole matter at our next annual meeting in Baltimore."

On motion of Dr. A. F. Bignon the Association adjourned to meet in Baltimore on the last Tuesday in July, 1873.

---

